# Low-dose emapalumab treatment in refractory macrophage activation syndrome secondary to adult onset still’s disease/systemic lupus erythematosus: insights from nine cases

**DOI:** 10.3389/fimmu.2025.1676749

**Published:** 2025-10-14

**Authors:** Jie Chen, Liling Zhao, Yanwei Lin, Xinyue Lian, Haiting Wang, Liyang Gu, Ran Wang, Xiaodong Wang, Shuang Ye, Qiong Fu

**Affiliations:** Department of Rheumatology, Ren Ji Hospital, Shanghai Jiao Tong University School of Medicine, Shanghai, China

**Keywords:** refractory macrophage activation syndrome, emapalumab, salvage treatment, efficacy, safety

## Abstract

**Objective:**

Macrophage activation syndrome (MAS) is frequently secondary to rheumatic diseases, with features including a cytokine storm and hemophagocytosis. Emapalumab is a monoclonal antibody that targets interferon-γ and has the ability to precisely regulate cytokines. This study aimed to investigate the efficacy and safety of low-dose emapalumab for patients with refractory MAS in the Chinese population.

**Methods:**

From January 2022 to July 2024, 9 patients with MAS secondary to adult-onset Still’s disease (AOSD) or systemic lupus erythematosus (SLE) received low-dose emapalumab following no response to prior conventional therapies. The laboratory parameters, therapeutic response, and safety were assessed following low-dose emapalumab-based treatment.

**Results:**

Of the nine MAS patients, 5 patients were secondary to AOSD and 4 patients were secondary to SLE. The overall response rate was 66.7% (6/9), 77.8% (7/9), 88.9% (8/9) and 88.9% (8/9) at week 1, 2, 4 and week 8, respectively. At the end of the follow-up period, up to 88.9% (8/9) of patients achieved complete remission. All patients demonstrated improvement or normalization of clinical manifestations and laboratory parameters. Notably, the median prednisone-equivalent dose for the patients was reduced by 85.5% during the treatment. Cytomegalovirus infection occurred in 33.9% (3/9) of patients, with no occurrence of serious adverse events reported.

**Conclusion:**

Our findings suggest that low-dose emapalumab may be a promising salvage option for refractory MAS in the Chinese population, but confirmation in larger prospective studies is required.

## 1 Introduction

Macrophage activation syndrome (MAS), also known as secondary hemophagocytic lymphohistiocytosis (HLH), is a rare and life-threatening systemic inflammatory disorder (1). MAS is most commonly secondary to rheumatic diseases, such as systemic juvenile idiopathic arthritis (sJIA), adult-onset Still’s disease (AOSD), and systemic lupus erythematosus (SLE), which is different from primary HLH caused by gene defects (2). This pathological condition is distinguished by inappropriate and sustained activation of cytotoxic cells and myeloid cells, resulting in a systemic cytokine storm including interferon-γ (IFN-γ), interleukin-18 (IL-18) and tumor necrosis factor-α (TNF-α), as well as IL-6, IL-1 and others (3). The 2022 EULAR/ACR Points to Consider for the Diagnosis and Management of HLH/MAS identified three categories of contributors involved in the development of HLH/MAS: genetic causes; predisposing conditions such as sJIA, lymphoma, and certain metabolic disorders that heighten susceptibility; and acute triggers including infections and immunotherapies (4). The clinical manifestations of MAS generally encompass persistent fever, rash, thrombocytopenia, anemia, and abnormal liver function. This particular situation has the potential to rapidly evolve, resulting in a multitude of organ dysfunctions and, consequently, elevated mortality rates (4, 5). Research has demonstrated that the 90-day all-cause mortality rate for adult patients afflicted with rheumatic disease-related MAS is 22.9% (6). In terms of early recognition and diagnosis, it is recommended to check ferritin levels when HLH/MAS is suspected, and to consider other indicators of inflammation, coagulation, or organ damage (4).

At present, there are no standard recommendations for the management of adult MAS, but rather predicated on clinical experience or reference to treatment employed in other forms of HLH. These therapeutic regimens principally comprise glucocorticoid shock therapy, cyclosporine and etoposide (7). However, 64% of MAS cases did not achieve remission through glucocorticoid pulse therapy, while 24% of cases did not respond to dexamethasone plus etoposide treatment, which further reflects the severe treatment dilemma (8, 9). Targeted cytokine inhibition therapies, including anakinra and tocilizumab, have emerged as potential new treatments for MAS (10). Additionally, studies have reported the use of JAK inhibitors, such as ruxolitinib and tofacitinib, for treating refractory AOSD complicated by MAS (11, 12). However, their efficacy still requires validation through larger-scale cohort studies (13). Furthermore, despite such advances, there remain a proportion of patients with MAS who do not achieve optimum treatment outcomes after initial treatment, highlighting the necessity of seeking effective remedial therapies (8).

Emapalumab is a fully human immunoglobulin G1 monoclonal antibody that has been shown to target the key cytokine IFN-γ in the pathogenesis of MAS. Emapalumab was approved by the FDA in November 2018, becoming the first drug globally used for the treatment of primary HLH (14). Mechanistically, it binds to IFN-γ and plays an effective role in neutralizing IFN-γ. Numerous studies conducted abroad have confirmed the efficacy and safety of emapalumab in patients suffering from MAS (2, 15, 16). In a single-arm, open-label, phase 2 trial conducted in five sites in Italy, France, Spain, the UK and the USA, emapalumab achieved MAS remission in almost all patients with MAS complicating sJIA/AOSD (2). However, there is a paucity of experience reports on the use of emapalumab in the Chinese population for MAS, especially for the refractory case. Given the high cost of emapalumab and the significant burden it may place on healthcare systems and patients, understanding its cost-effectiveness is crucial. In the context of limited healthcare resources, it is essential to evaluate whether the benefits of emapalumab justify its costs, particularly in treating refractory MAS cases. Herein, we present a cohort of patients with refractory MAS who have previously failed to traditional treatment, aiming to evaluate the efficacy and safety of low-dose emapalumab in Chinese adult refractory MAS patients, as well as to provide preliminary insights into its potential cost-effectiveness in this specific population.

## 2 Methods

### 2.1 Study design and patients

This retrospective case series study included adult patients with refractory MAS who received salvage emapalumab therapy in the Department of Rheumatology and Immunology, Renji Hospital, Shanghai Jiao Tong University School of Medicine, Shanghai, China, from January 2022 to July 2024. All patients included in the study were prospectively identified and met the diagnostic criteria for MAS based on the HLH-2004 criteria. In a subset of patients with prior history of MAS, the HScore was applied for diagnostic evaluation. To address the concern about selection bias, all consecutive eligible patients were included in this analysis. They received emapalumab following no clinical responses to prior treatments, including glucocorticoids, etoposide, cyclosporine, or JAK inhibitors. The study protocol was approved by the Ethics Committee of Renji Hospital and was conducted in accordance with the ethical principles outlined in the Declaration of Helsinki (LY2025-170-A).

### 2.2 Treatment

The therapeutic dose of emapalumab was 1 mg/kg referring to a previous trial. Frequency could be adjusted on investigator’s assessment of response, with a median number of infusions was 3 (range: 1-5). Other drugs are also permitted, depending on the patient’s condition. Glucocorticoid tapering could be initiated as soon as the patients’ conditions allowed based on investigator’s assessment.

Medical records were systematically reviewed by three independent rheumatologists. Demographic characteristics, clinical presentation (such as fever, rash, lymphadenopathy and organ enlargement), laboratory parameters (including white blood cell [WBC], neutrophil, platelet counts, hemoglobin, ferritin levels, fibrinogen, D-Dimer, soluble CD25 [sCD25], triglyceride, and liver function), therapeutic interventions, clinical outcomes and follow-up evaluations of patients were collected. Given the dynamic nature of disease and the necessity for close monitoring, the reassessment of MAS-related indicators was meticulously scheduled to occur every 2 to 7 days until the patient was discharged. Before the start of treatment, we carried out a comprehensive virus screening for all patients, through PCR detection and serological detection.

### 2.3 Efficacy and safety

Following completion of the emapalumab treatment, the efficacy of emapalumab was evaluated based on clinical symptoms, laboratory parameters and established standards (NCT03311854/NCT05001737) (2). Treatment response was categorized into three distinct classifications: (1) complete remission (CR), defined as full normalization of all clinical and laboratory parameters; (2) partial remission (PR), defined as improvement of at least two parameters or symptoms by ≥25%, as determined by the attending physician, with specific thresholds requiring: a ≥25% reduction in sCD25, SF, and TG; absence of blood transfusion dependence; an increase of ≥100% in neutrophil count if the baseline was <0.5 × 10^9^/L, or an increase of ≥100% returning to normal if baseline neutrophils were 0.5-2.0 × 10^9^/L; and a ≥50% reduction in alanine aminotransferase (ALT) levels for patients with ALT >400 U/L; and (3) no response (NR), defined as failure to meet the aforementioned criteria for CR or PR. The overall response rate (ORR) was defined as the proportion of patients achieving either CR or PR (17). Meanwhile, the safety of emapalumab was assessed based on the adverse events (AEs) that occurred during the treatment and follow-up period.

### 2.4 Statistical analysis

Data from all nine patients were subjected to descriptive statistical analysis, and no form of statistical comparison was performed. Categorical variables are presented with the number and percentage within each category. Continuous variables are reported as median (range).

## 3 Results

### 3.1 Demographics and clinical characteristics

Between January 2022 and July 2024, a total of 9 patients with refractory MAS were enrolled in this analysis. **Table 1** summarized the baseline characteristics of the patients before emapalumab treatment. Among the 9 patients treated with emapalumab, 77.8% were female (n = 7), with a median age of 38 years (range: 18–56 years). Of the nine MAS patients, 5 patients were secondary to AOSD and 4 patients were secondary to SLE. All patients exhibited fever prior to emapalumab treatment except for patient 3. Splenomegaly was observed in 22.2% (2/9) of cases, while myelophagocytosis was observed in 44.4% (4/9) of cases. Furthermore, the activity of natural killer (NK) cells was evaluable in 4 patients, with levels of 0.62%, 0.90%, 3.83%, and 12.42% observed in patients 1, 2, 3, and 4, respectively. Prior to the administration of emapalumab, all nine patients had undergone immunosuppressive therapy (**Table 2**), including glucocorticoids (n = 9), etoposide (n = 4), and cyclosporine (n = 4). Of the nine patients, 6 received JAK inhibitor treatment, including ruxolitinib (n=5) and tofacitinib (n=1). These patients have shown an inadequate response to the initial or re-intensified MAS-directed therapy or an inability to tolerate full-dose re-intensification of etoposide-based therapy.

**Table 1:** Baseline characteristics of emapalumab-treated patients with MAS.

Characteristics	Patient 1	Patient 2	Patient 3	Patient 4	Patient 5	Patient 6	Patient 7	Patient 8	Patient 9
Gender	Male	Male	Female	Female	Female	Female	Female	Female	Female
Age, years	41	31	38	56	56	18	38	37	18
Underlying disease	AOSD	AOSD	AOSD	AOSD	AOSD	SLE	SLE	SLE	SLE
Febrile	yes	yes	no	yes	yes	yes	yes	yes	yes
Splenomegaly	no	no	no	no	yes	no	yes	no	no
WBC, ×10^9/L	1.34	0.94	0.91	0.93	6.07	4.91	5.37	3.42	1.86
Neutrophil count, ×10^9/L	0.78	0.84	0.66	0.53	5.4	3.86	5.04	4.01	0.85
Hemoglobin, g/L	100	52	61	111	79	103	76	92	119
Platelet count, ×10^9/L	177	87	91	38	241	92	36	177	86
Triglycerides, mmol/L	1.7	2.86	2.52	1.78	2.76	1.8	1.57	1.2	1.85
Fibrinogen, g/L	1.59	0.88	0.65	1.98	1.74	1.71	1.73	1.95	4.64
Ferritin, ng/mL	145319	28160	13583	18219.6	2919	2772	357	3355	3075.8
NK cell activity, %	0.62%	0.9%	3.83%	12.42%	NE	NE	NE	NE	NE
sCD25 (sIL-2R), pg/mL	4332.45	2110.19	1303.25	1332	527.8	NE	791.68	1114.22	1532
Hemophagocytosis in bone marrow	yes	no	yes	no	yes	no	no	no	no
ESR, mm/h	58	34	26	31	44	12	16	6	74
CRP, mg/L	14.15	1.12	6.11	29.56	1.77	4.3	9.5	1.94	44.81
ALT, U/L	433	104	173	63	23	144	35	57	61
AST, U/L	418	90	39	51	38	68	28	59	69
LDH, U/L	2062	1366	792	348	874	732	555	635	838
H score^#^	181	212	212	216	196	152	172	175	173
MS score^&^	14.13	3.57	1.62	1.26	-0.25	0.05	-0.21	-1.64	-0.97

^#^The H score can be used to estimate an individual’s risk of developing reactive hemophagocytic syndrome. This scoring system is available online, http://saintantoine.aphp.fr/score/ (Fardet L, Galicier L, Lambotte O, et al. Development and validation of the HScore, a score for the diagnosis of reactive hemophagocytic syndrome. Arthritis Rheumatol 2014; 66:2613–20).

^&^The MS score has been developed for evaluating MAS in systemic juvenile idiopathic arthritis and may also be useful in adult MAS, including patients with AOSD-associated MAS (Wang R, Li T, Ye S, et al. Application of MS score in macrophage activation syndrome patients associated with adult-onset Still’s disease. Ann Rheum Dis 2021;80:e145).

AOSD, adult-onset Still’s disease; WBC, white blood cell; sCD25, soluble CD25; ESR, erythrocyte sedimentation rate; CRP, C reactive protein; ALT, alanine aminotransferase; AST, aspartate aminotransferase; LDH, lactate dehydrogenase; NE, not evaluated.

**Table 2:** Previous therapies prior to emapalumab treatment.

Previous therapies	Patient 1	Patient 2	Patient 3	Patient 4	Patient 5	Patient 6	Patient 7	Patient 8	Patient 9
Treatment for MAS before emapalumab	GCS, etoposide, cyclosporine, ruxolitinib	GCS, etoposide	GCS, etoposide, cyclosporine, ruxolitinib	GCS, tofacitinib	GCS, cyclosporine, ruxolitinib	GCS, ruxolitinib	GCS	GCS, etoposide, cyclosporine	GCS, ruxolitinib
Glucocorticoids equivalent to prednisone prior to emapalumab, mg/kg/day	2.27	3.20	3.80	2.53	1.13	1.87	4.47	1.33	1.87
Duration between MAS recurrence and emapalumab, weeks	3.5	1	1	1	1	1	2	4	0.5

GCS, glucocorticoids.

### 3.2 Efficacy and safety of emapalumab

**Figure 1:**
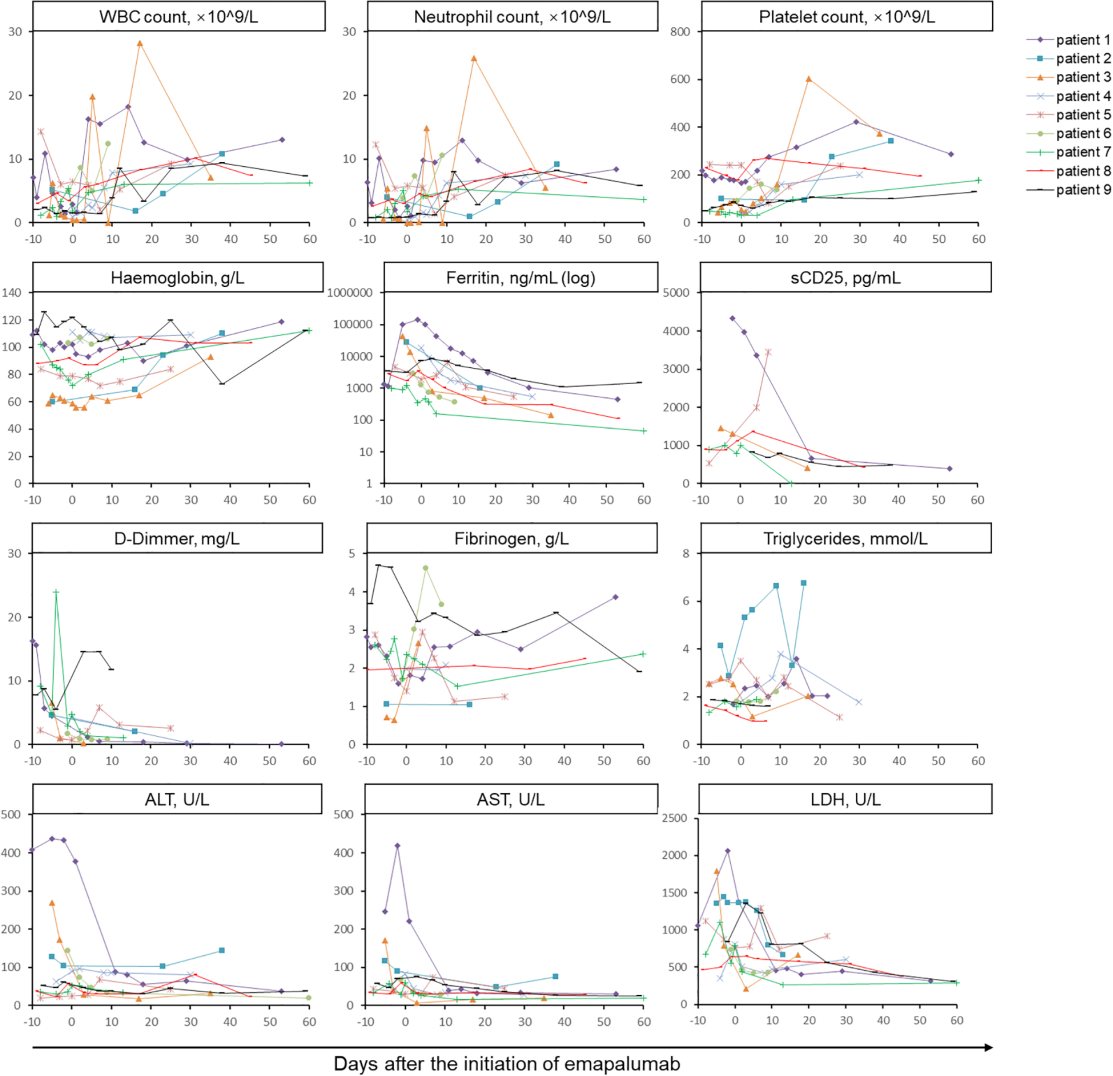
Longitudinal monitoring of laboratory parameters associated with MAS in nine patients treated with emapalumab. The horizontal axis represents the days after the initiation of emapalumab and the vertical axis represents levels of laboratory parameters. The assessment of MAS-related indicators was conducted every 2 to 7 days until the patient was discharged. Data were subjected to descriptive statistical analysis, and no form of statistical comparison was performed. WBC, white blood cell; sCD25, soluble CD25; ALT, alanine aminotransferase; AST, aspartate aminotransferase; LDH, lactate dehydrogenase.

Within a few days of commencing treatment with low-dose emapalumab, body temperature returned to normal in 88.9% (8/9) of patients. Further, patient 5 demonstrated persistent low-grade fever, which subsequently returned to normal during subsequent follow-up. Prior to the administration of emapalumab, 4 patients exhibited persistent rashes, which subsequently resolved following the treatment of emapalumab. During a median follow-up period of 81 days (range: 64–301 days), laboratory parameters associated with MAS showed improvement or normalization (**Figure 1**). During the follow-up period, WBC, neutrophil, and platelet counts initially increased after treatment of emapalumab, and were subsequently normalized in 9/9 (100%), 9/9 (100%), and 7/9 (77.8%) patients, respectively. Hemoglobin levels returned to normal in 4/9 patients (44.4%). Ferritin levels gradually decreased, with 6/9 (66.7%) patients returning to normal levels. Fibrinogen returned to normal in 7/8 (85.7%) patients and the levels of D-Dimer decreased overall. Among 6 patients with continuously evaluated sCD25, the levels of sCD25 decreased in 5 patients after emapalumab, except for 1 patient with an increased level of sCD25. The levels of triglyceride gradually decreased after the initiation of emapalumab. Liver function tests showed that ALT and aspartate aminotransferase (AST) returned to normal in 8/9 (88.9%) and 9/9 (100%) patients, respectively. Although lactate dehydrogenase (LDH) did not return to normal, a decreasing trend was observed.

The earliest clinical response was observed at 3 days after emapalumab. **Table 3** illustrated the changes in efficacy of emapalumab in patients with refractory MAS within a period of 8 weeks. At the first week after initiation of emapalumab, 6 (66.7%) patients achieved PR, while 3 patients (33.3%) showed NR, with an ORR of 66.7%. By week 2, 5 (55.6%) patients achieved PR, and 2 (22.2%) patients achieved CR, yielding an ORR of 77.8%. By week 4, 5 (55.6%) patients achieved CR and 3 (33.3%) patients achieved PR, with an ORR of 88.9%. By week 8, 88.9% (8/9) of patients achieved CR, with an ORR of 88.9%. Only one patient failed to achieve PR, but both the clinical manifestations and laboratory parameters improved. However, during the tapering of corticosteroids, MAS relapsed. Cytomegalovirus (CMV) infection was observed in 3 patients during treatment and was solved using standard antiviral treatments. No unexpected AEs were observed during the treatment of emapalumab, and all of AEs were grade 1. No serious AEs (SAEs) were documented, and all patients were alive at the last visit.

**Table 3:** The therapeutic dose and therapeutic response of emapalumab for the enrolled patients.

Therapeutic Regimean	Patient 1	Patient 2	Patient 3	Patient 4	Patient 5	Patient 6	Patient 7	Patient 8	Patient 9
Number of emapalumab infusions	5	3	2	3	1	4	2	3	4
Combined medications	Cyclosporine, ruxolitinib	Ruxolitinib	Cyclosporine, ruxolitinib	Ruxolitinib	Cyclosporine, ruxolitinib	HCQ	Cyclosporine	Cyclosporine, ruxolitinib	Tacrolimus, baricitinib
Duration of follow-up after emapalumab start, days	81	301	110	253	77	64	60	45	98
Patient’s response									
At week 1	PR	NR	PR	PR	NR	NR	PR	PR	PR
At week 2	CR	NR	PR	PR	PR	NR	CR	PR	PR
At week 4	CR	PR	CR	PR	PR	NR	CR	CR	CR
At week 8	CR	CR	CR	CR	CR	NR	CR	CR	CR
Survival	yes	yes	yes	yes	yes	yes	yes	yes	yes
Glucocorticoids equivalent to prednisone at last follow-up, mg/kg/day	0.33	0.07	0.40	0.07	0.87	0.53	0.20	0.60	1.27

HCQ, Hydroxychloroquine; CR, complete remission; PR, partial remission; NR, no response.

### 3.3 Glucocorticoid-sparing effect

**Figure 2:**
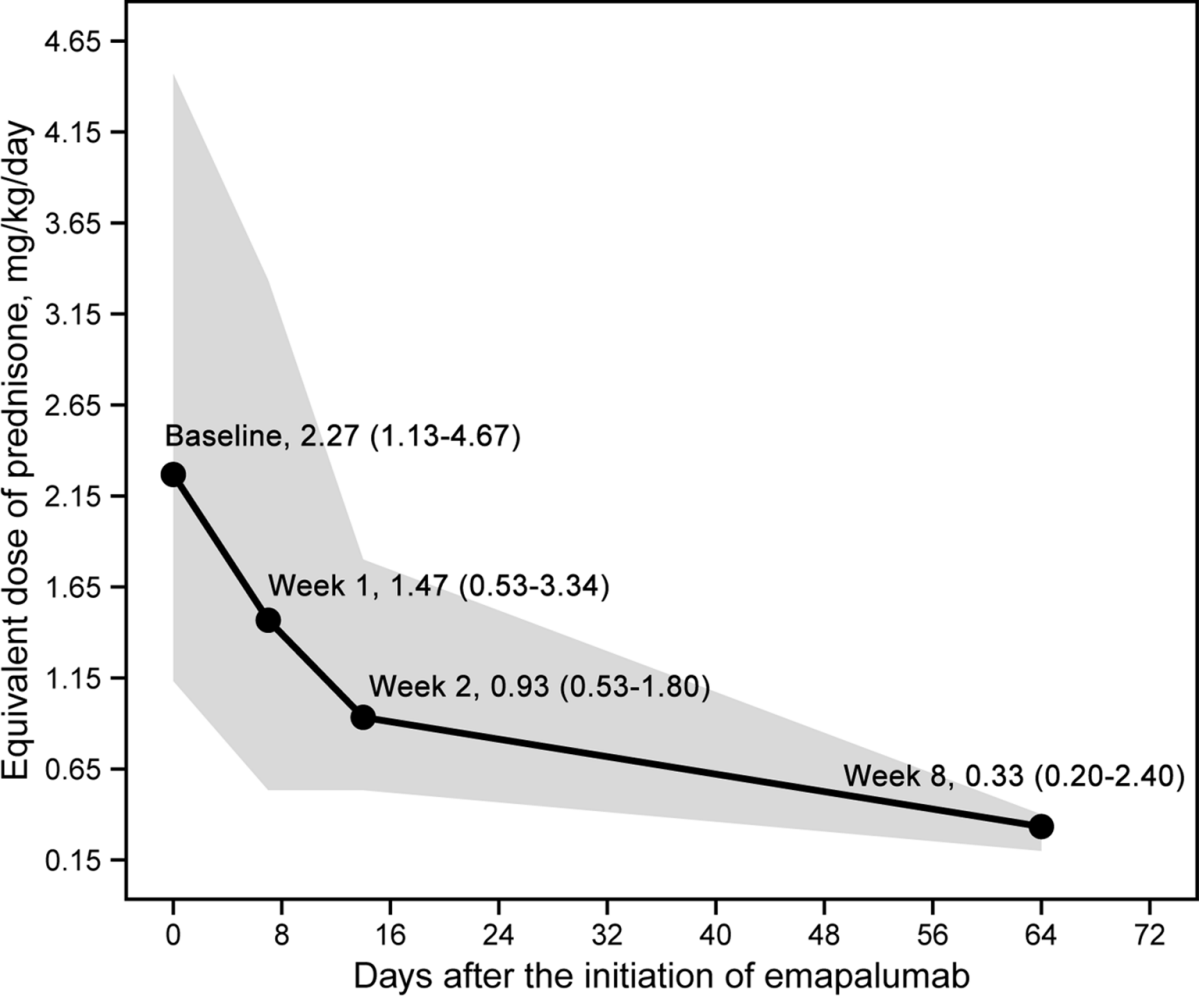
Daily dose changes of glucocorticoids (prednisone equivalent dose) after the initiation of emapalumab.

An overall reduction in the prednisone-equivalent dose was observed during treatment, which is shown in **Figure 2**. A substantial decrease in prednisone-equivalent dose was observed as early as week 2. Over the course of 8 weeks, the median prednisone-equivalent dose was reduced by 85.5%, from 2.27 mg/kg/day (range: 1.13 - 4.47) at the commencement of emapalumab treatment to 0.33 mg/kg/day (range: 0.20 - 0.40) at week 8.

**Figure 3:**
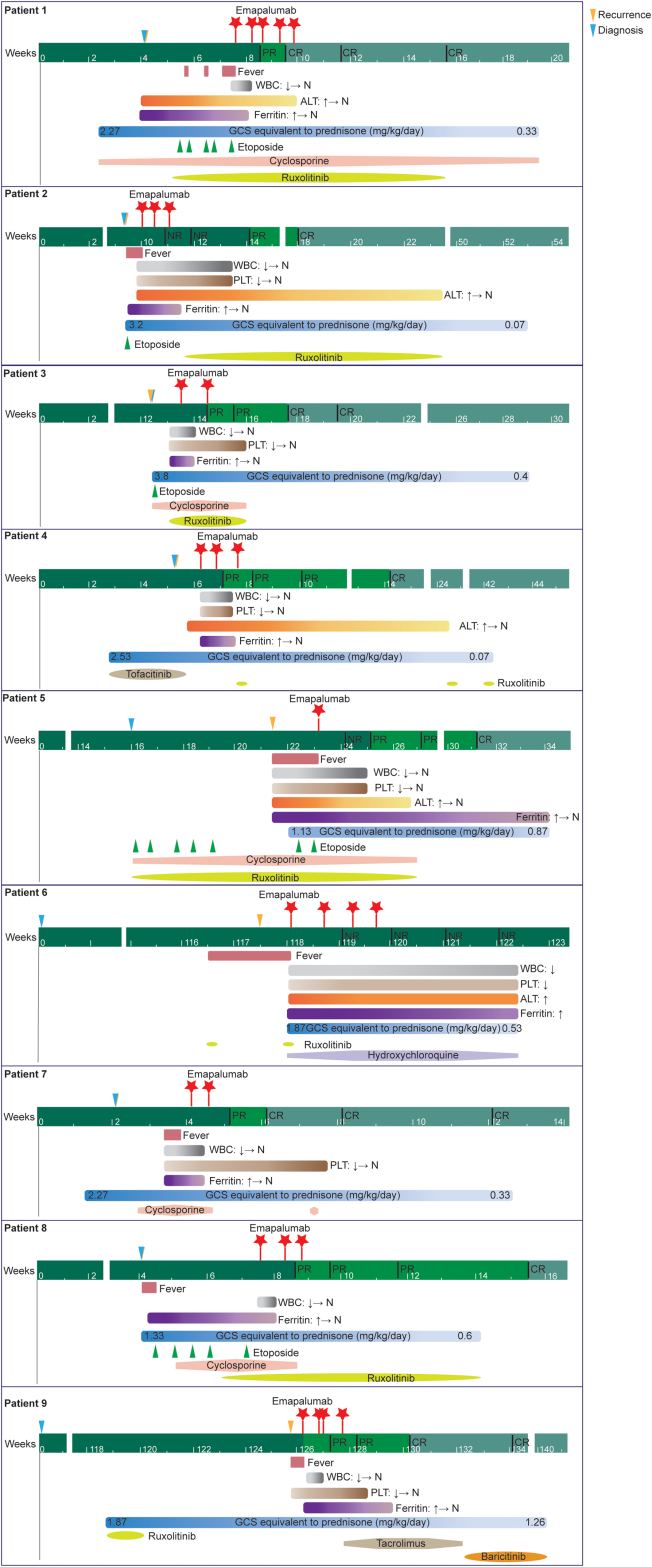
A detailed timeline chart for each patient illustrating clinical manifestations and therapies.

A detailed timeline chart for each patient illustrating clinical manifestations and therapies was presented in **Figure 3**.

## 4 Discussion

Refractory MAS patients still have a significant unmet medical need, although some progress has been made in biologic therapy. This study constitutes the inaugural report on the utilization of low-dose emapalumab in China, thereby substantiating the finding that low-dose emapalumab not only accomplishes satisfactory clinical efficacy but concomitantly engenders substantial improvement in systemic symptoms, reduction in glucocorticoid dose, and maintenance of controllable safety. Low-dose emapalumab treatment may have the capacity to address the clinical requirements for refractory MAS patients, with outcomes that are both timely and effective.

Emapalumab was predominantly used to treat refractory and recurrent disease. The majority of patients had received HLH-related therapies before (>50%) and concurrently (100%) with emapalumab, which aligns with its role as a second-line therapy for patients with primary HLH. Glucocorticoids and anakinra were the most commonly used treatments. In line with these findings, retrospective studies have demonstrated that HLH is most often managed with pulse therapy involving high-dose glucocorticoids (either intravenous methylprednisolone or oral prednisolone), cyclosporine, or anakinra (18–21). The majority of concomitant medications were initiated prior to emapalumab treatment, largely reflecting an inadequate response to prior therapies. In this setting, emapalumab was used as an add-on strategy. After the introduction of emapalumab, clinical and laboratory parameters improved, avoiding the need for increased glucocorticoid doses and allowing for successful dose reduction. In some patients, ruxolitinib was initiated later as a sequel therapy (patient 2, patient 4 and patient 9) to consolidate the response and maintain clinical improvement.

In the instance of excessive activation and proliferation of T lymphocytes and macrophages, the manifestation of MAS is typically characterized by a series of clinical symptoms and signs, including fever and rash, as well as a range of laboratory parameter abnormalities (15). Our findings have demonstrated that low-dose emapalumab-based treatment exerts a significant positive effect on the clinical and laboratory characteristics of patients with MAS among the Chinese population. Within a few days of commencing treatment with low-dose emapalumab-based strategy, the body temperature of almost all patients returned to normal. During the median follow-up period, key laboratory parameters related to MAS improved or returned to normal. Similarly, in several retrospective studies, patients with MAS treated with high-dose corticosteroids, cyclosporine, anakinra, or ruxolitinib showed improvements in laboratory parameters such as ALT, AST, and normalization of clinical signs, indicating MAS remission (21–24). The efficacy measure of this study, known as the ORR, was referred to established standards that combine resolution of clinical signs and symptoms and of the abnormalities in MAS laboratory parameters (2). The initial phase of treatment, spanning the first few days to one to two weeks, is of paramount importance for the evaluation of the response to treatment. This study evaluated the treatment response of MAS patients during weeks 1, 2 and 8 of emapalumab treatment. It is noteworthy that the initial PR occurred on the third day, indicating a rapid response in patients with refractory MAS (13). By week 8, 88.9% (8/9) of refractory MAS patients achieved CR, which is comparable to the previous remission rate of 93% (13/14) of emapalumab in the multinational prospective pilot trial (2). Recent studies of ruxolitinib demonstrate that the ORR of as salvage treatment in adult MAS is 80% (16/20) - 87.5% (7/8), which is similar to the remission rate in this study (13, 25). Several studies reported the efficacy of IL-1 blocker anakinra in pediatric patients with sJIA/MAS, with CR rates ranging from 50% to 100% (23, 26–28). For canakinumab, another IL-1 blocker, 87.5% (7/8) patients achieved a CR (29).

A substantial decrease in prednisone-equivalent dose was observed as early as week 2. Notably, the median prednisone-equivalent dose was reduced by 85.5%, from 2.27 mg/kg/day (range: 1.13 - 4.47) at the commencement of emapalumab treatment to 0.33 mg/kg/day (range: 0.20 - 0.40) at week 8, suggesting that emapalumab may result in a gradual reduction in glucocorticoid levels, and thereby contribute to a reduction in drug-related toxicity within this demographic (30). Our findings mirrored the approximately 96% reduction in median average daily glucocorticoid dose that was observed with emapalumab in a prospective trial involving patients with sJIA/AOSD, who had an inadequate response to high-dose glucocorticoids (2).

Our population consisted of patients with refractory/recurrent disease at high risk of mortality; however, none of the patients died (overall survival: 100%). In a real-world study for rheumatologic disease-associated HLH, the 12-month survival probability from emapalumab initiation was 86.7% and 90.0% in the subset with sJIA/AOSD (30). Some retrospective studies have also demonstrated good prognosis in patients with rheumatologic disease-associated HLH with mortality rates ranging from 8.3% to 28.6% (18, 20, 22, 31). The higher survival rate observed in our study compared to previous studies may be attributable to the limited sample size, which could have led to an overestimation of the survival rate. However, it cannot be denied that, despite variations in survival rates across different studies, patients treated with emapalumab consistently demonstrated favorable prognosis. Higher overall survival of emapalumab also appears to be higher in comparison to other therapies. In Eloseily et al. study, anakinra treatment of pediatric patients with secondary HLH/MAS was associated with improved overall survival (73%) (32). In Henter et al. the survival rate of etoposide-based protocols was 55% (33). Etoposide, considered only for treating refractory disease or central nervous system involvement, may be associated with myelosuppression and risk of secondary infections and malignancies (34). This explains its relatively lower survival benefit. IL-6 blockers (e.g., tocilizumab) are prescribed with caution because they may mask the clinical symptoms of sJIA/MAS (35). In Kim et al. study, the tocilizumab group had a significantly lower 8-week survival rate (12.5% vs. 51.9%) and a significantly increased risk of death compared to conventional treatments (36).

Patients received emapalumab-containing combination therapy. Although the benefits to patients cannot be entirely attributed to emapalumab, the primary benefits still stem from emapalumab, which was beyond doubt. The evidence comes from the fact that the enrolled patient received glucocorticoids, etoposide, cyclosporine, or JAK inhibitors prior to receiving emapalumab, but all had recurrent fever. Collectively, the above results indicate that low-dose emapalumab can achieve ideal therapeutic effects in Chinese patients with refractory MAS and is a promising salvage treatment strategy. However, due to the limitations of its retrospective nature and small sample size, the study results are still in the preliminary stage and are only used to generate hypotheses. These data should be interpreted with caution.

Current guidelines and clinical experience provide limited data on the most effective and safe dosing regimens for this specific condition. Of course, optimization of emapalumab dose and dosing frequency based on prospective trials and clinical evidence was still an unmet need. Here, the therapeutic dose of emapalumab in our study was 1 mg/kg, was lower than those in the open-label, prospective trial of emapalumab in patients with rheumatologic disease-associated HLH and underlying sJIA/AOSD (6 mg/kg) (2). In a retrospective medical chart review study, the median (range) of emapalumab starting dose in the subset of patients with sJIA/AOSD was 3.7 [0.9-5.9] mg/kg (30). This variability underscores the significant differences in individual patient responses and the need for personalized dosing strategies. Given the high price of emapalumab in China, which can be a significant barrier to its widespread use, we explored the feasibility and efficacy of lower doses in our patient population.

As the efficacy of other diagnostic tools has not been extensively validated in the Chinese population, the diagnosis of MAS in clinical practice still relies on the HLH-2004 classification criteria. Furthermore, studies have demonstrated that HScore and MS Score exhibit high sensitivity and specificity in the diagnosis of MAS in adult patients with rheumatoid arthritis, thus underscoring their value as clinical tools (37). Consequently, this study also referred to HScore and MS Score during the diagnosis process, thereby enhancing the accuracy of diagnosis. CXCL9, a chemokine which is specifically induced by IFN-γ, was used as a biomarker for the global activity of IFN-γ in previous trial (38). Buatois et al. showed that CXCL9 is associated with disease severity in secondary HLH (39). Chandrakasan et al. found that levels of IFN-γ and CXCL9 were higher in patients with CTD-MAS and returned to normal after MAS resolution (30). Emapalumab, is expected to reduce CXCL9 levels, forming a clear pharmacodynamic mechanism. Jacqmin et al. proposed a CXCL9 threshold related to treatment response (around 300 pg/mL) (38), this mainly comes from model derivation and retrospective analysis, and still needs to be validated in prospective studies before it can be used for clinical decision-making. Here, in patients 3, 5, and 7, the levels of IFN-γ, before administration of emapalumab, were 4.64 pg/mL, 2.59 pg/mL, and 7.44 pg/mL, respectively, while the levels of CXCL9 were 2152.52 pg/mL, 4606.9 pg/mL, and 13421 pg/mL, respectively. Although only a limited number of cytokine test results were collected, our finding suggested that high levels of CXCL9 can occur despite insignificantly elevated levels of IFN-γ, indicating that emapalumab may be more effective in cases of CXCL9 activation. Therefore, while existing studies have suggested a correlation between CXCL9 and IFN-γ, this remains an exploratory analysis. The IFN-γ-related inflammatory status in MAS/HLH patients may need to be considered in relation to CXCL9, but whether the timing of emapalumab administration should be determined based on CXCL9 requires further validation through clinical trials. Furthermore, the safety profile of emapalumab observed in this study was favorable, with no occurrence of SAEs reported. In the viral screening before treatment, we found that some patients had low-level Epstein-Barr virus and CMV infections, and all these patients had received antiviral therapy before treatment. During the treatment, we did not observe any new viral activity or significant increase in viral load in any patients. 33.3% of patients of the patients experienced mild symptoms of CMV infection, which were resolved with standard antiviral therapy. The essence of MAS is the loss of immune regulation, manifested as the dysfunction of NK cells and cytotoxic T cells, which cannot effectively eliminate virus-infected cells (40). It is worth noting that in the prospective trial, emapalumab treatment for MAS reported SAEs of CMV infections, which were considered to be caused by long-term use of high-dose glucocorticoids for immunosuppression in critically ill patients (2). Similarly, there have been reports of CMV infection in 20% (4/20) of adverse reactions associated with ruxolitinib in the treatment of adult refractory rheumatoid arthritis-related MAS. Consequently, further research was required to ascertain the underlying causes of CMV infection in MAS patients. Overall, in this study, we strictly followed routine viral monitoring and promptly took corresponding measures based on the monitoring results, ensuring the safety and efficacy of the treatment. We will further optimize viral monitoring strategies in subsequent studies to provide patients with safer and more effective treatment plans.

This study has several limitations. Firstly, as a single-center, small sample size retrospective case series study, the data collection in this study is based on previous medical records, and the enrollment process was influenced by factors including the cost of medications and patients’ medical insurance, which may result in information bias and selection bias. Secondly, due to the lack of standard treatment protocols for MAS patients after failure of high-dose glucocorticoid therapy, there are currently no comparable drugs available as a control group for direct comparison. Thirdly, in this study, the dosage of emapalumab used was all 1 mg/kg, which was based on the approved instructions. However, previous prospective clinical trials utilized an initial dose of 6 mg/kg and a subsequent dose of 3 mg/kg (2). Consequently, further study is required to ascertain the optimal dose of emapalumab for the treatment of MAS in the Chinese population. Fourthly, due to patients received MAS-related therapies concurrently with emapalumab, the efficacy of emapalumab alone may be compromised. Establishing different treatment groups to evaluate the effects of monotherapy and combination therapy separately, thereby more accurately assessing the role of each drug, could be considered. Additionally, pharmacokinetic and pharmacodynamic studies could be conducted to further explore the mechanisms of drug-drug interactions, providing a basis for optimizing treatment regimens. Finally, due to medical expenses, our patients usually do not use emapalumab for a long time, so the long-term efficacy of emapalumab is limited. Regardless, what is more meaningful is that low-dose emapalumab can help patients achieve rapid disease remission, allowing them to proceed with subsequent treatments, especially when they cannot obtain adequate response from conventional therapies. Future prospective studies with larger sample sizes are needed in the Chinese population to further elucidate the long-term efficacy and safety profile of low-dose emapalumab in refractory MAS through standardized trial designs.

In conclusion, the present study provides preliminary evidence to suggest that low-dose emapalumab-based treatment is rapid, efficacious and safe for use in Chinese adult MAS patients.

## Data Availability

The original contributions presented in the study are included in the article/supplementary material. Further inquiries can be directed to the corresponding authors.
